# Microbial production of mevalonate by recombinant *Escherichia coli* using acetic acid as a carbon source

**DOI:** 10.1080/21655979.2017.1323592

**Published:** 2017-06-02

**Authors:** Xin Xu, Meng Xie, Qian Zhao, Mo Xian, Huizhou Liu

**Affiliations:** a CAS Key Laboratory of Biobased Materials, Qingdao Institute of Bioenergy and Bioprocess Technology, Chinese Academy of Sciences, Qingdao, China; b University of Chinese Academy of Sciences, Beijing, China; c College of Environment and Safety Engineering, Qingdao University of Science and Technology, Qingdao, China

**Keywords:** Acetate assimilation, aeration, mevalonate, two-stage fed-batch, *Escherichia coli*

## Abstract

We sought to produce mevalonate, an important organic acid, by recombinant *Escherichia coli*, using acetic acid, a less costly alternative feedstock, as a carbon source. In this study, the mevalonate biosynthesis pathway originating with acetate was constructed in recombinant *E. coli*, resulting in the production of 1.06 g L^−1^ mevalonate with a productivity of 0.03 g L^−1^ h^−1^ in a 5-L bioreactor. The mevalonate concentration and productivity were significantly enhanced with increased cell density during 2-stage aerobic fermentation, reaching 7.85 g L^−1^ and 0.13 g L^−1^ h^−1^, respectively. Fed-batch fermentation was further optimized under anaerobic and microaerobic conditions, and mevalonate concentrations reached 3.05 g L^−1^ and 4.97 g L^−1^, respectively, indicating that the oxygen supply exerts a large impact on mevalonate production from acetate. This study describes a method with high potential to produce mevalonate with the engineered *E. coli* strain XU143 using the less costly alternative feedstock acetate as a carbon source.

## Introduction

Mevalonate (β-hydroxy-β-methyl-δ-valerolate) is a key organic compound that is produced via the mevalonate pathway in most eukaryotes and higher plants. This compound is the precursor for steroids and terpenes, including isoprene, pinene, carotenoid, artemisinin, and paclitaxel, which are widely used as material monomers, advanced fuels and pharmaceuticals. Mevalonate biosynthetic pathways originating from sugar have been constructed in recombinant *E. coli*.^1,2^ However, with global food shortages and increasing demand for mevalonate, non-food substrates have attracted attention as alternative feedstocks. Acetate is a potential low-cost carbon source because it is an ordinary, aqueous material produced by both biologic and chemical processes. (1) Specifically, acetate is a byproduct of anaerobic digestion processes such as acetogenesis, acidogenesis, and methanogenesis.^3,4^ (2) Additionally, biomass hydrolysates contain acetate because hemicelluloses are generally acetylated.^5^ (3) Acetate is also co-produced during syngas fermentation.^6^

**Figure 1.**
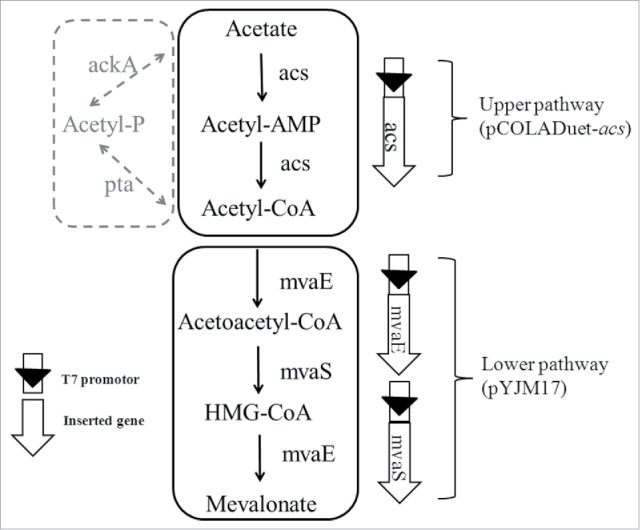
The metabolic pathways of mevalonate biosynthesis from acetate. Solid line: mevalonate biosynthesis pathway used in this study; dotted line: acetate assimilation pathway in *E. coli*. Genes in upper pathway: *acs*, for acetyl-CoA synthase; Genes in lower pathway: *mvaE*, for acetyl-CoA acetyltransferase; *mvaS*, for HMG-CoA synthase.

*Escherichia coli* is one of the most commonly used host microorganisms in the biochemical industry. Acetate metabolism pathways in *E. coli* have previously been elucidated. Acetate accumulates due to overflow metabolism when *E. coli* is cultivated on glucose, but acetate is also used as a primary carbon source during glucose starvation.^7^ In *E. coli*, acetate is converted into acetyl-CoA via the AMP-forming acetyl CoA synthetase (*acs*) and phosphotransacetylase/acetate kinase (*pta-ackA*) pathways (Fig. 1).^8^ Subsequently, acetyl-CoA enters the TCA cycle and glyoxylate cycle, providing energy and precursors for cell growth. Recently, free fatty acids were produced from acetate by an engineered *E. coli* strain, and the *acs* pathway was more effective than the *pta-ackA* pathway.^9^ *E. coli* also represents an attractive host to convert acetate into other chemicals, including mevalonate, through various biocatalysis processes. To our knowledge, there have been no previous reports describing mevalonate biosynthesis from acetate.

Central carbon metabolism in *E. coli* strains cultured with acetate differs greatly from glucose-based metabolism. During acetate assimilation, glycolytic flux and the pentose phosphate pathway (PPP) are almost completely impaired.^10,11^ However, certain important cofactors, such as ATP and NAD(P)H, are generated by both pathways when *E. coli is* cultivated on glucose.^12,13^ This may exert adverse effects on cell growth and product synthesis. Studies investigating fermentation strategies and conditions may address this problem. In this study, the acetate-derived mevalonate biosynthesis pathway was constructed in recombinant *E. coli*, which was cultured in a bioreactor using acetate as the only carbon source. Then, 2-stage fermentation for mevalonate production was performed to improve cell growth and mevalonate concentration. Additionally, mevalonate production and overall conversion from acetate under different aerobic conditions were compared.

## Result and discussion

### Construction of the mevalonate biosynthesis pathway originating with acetate

**Figure 2.**
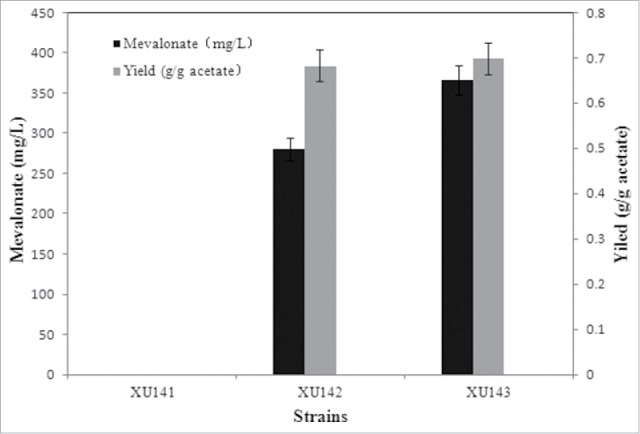
Mevalonate production of different strains using acetate as a carbon source when cultured in shaking flasks. When OD_600_ reached 0.6–0.9, cultures were induced for 36 h using IPTG in shake-flasks. All the experiments were performed in triplicates. 368 g L^−1^ mevalonate was obtaind from acetate with XU143, while the control strain XU141 failed to form mevalonate from acetate.

The AMP-forming acetyl CoA synthetase from *Escherichia coli*, which catalyzes acetate to acetyl-CoA (upper pathway), and the MVA pathway from *Enterococcus faecalis*, which converts acetyl-CoA into mevalonate (lower pathway) (Fig. 1), were assembled in *E. coli* strain XU143 to achieve efficient mevalonate production originating with acetate. This assembly was performed with the following considerations: first, AMP-forming acetyl CoA synthetase was previously verified to be more efficient for acetate assimilation.^8,9^ Second, the MVA pathway from *E. faecalis* was shown to be efficient in our previous study.^2^ After shake-flask cultivation for 48 h in the presence of acetate, 368 g L^−1^ mevalonate was obtained from *E. coli* strain XU143, while the control strain XU141 failed to form mevalonate. The mevalonate production efficiency in strain XU143 was approximately 1.3-fold greater than that of strain XU142, which only contained the lower pathway (280 g L^−1^) (Fig. 2). This result indicates acetate-derived mevalonate biosynthesis by XU143 has much developmental potential.

### Fed-batch fermentation using acetate as the sole carbon source (FB-1)

**Figure 3.**
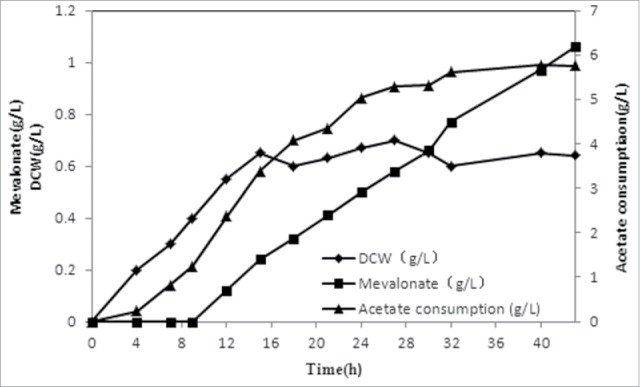
Fed-batch fermentation using acetate as the sole carbon source. Induction was performed at 9 h at the DCW of 0.4 g L^−1^. The maximum concentration of mevalonate reached 1.06 g L^−1^. Overall yield and productivity were 0.30 g g^−1^ and 0.03 g L^−1^ h^−1^, respectively.

As shown in Figure 2, mevalonate production by XU143 using acetate as the sole carbon source was achieved. Next, we grew XU143 in a bioreactor to obtain larger amounts of mevalonate. pH-coupled fed-batch fermentation was performed with XU143 using acetate as the sole carbon source. Biomass, mevalonate accumulation and acetate consumption were monitored over the course of fermentation (Fig. 3). The mevalonate concentration peaked at 1.06 g L^−1^ after 43.5 h of cultivation. Overall yield and productivity were 0.30 g g^−1^ and 0.03 g L^−1^ h^−1^, respectively. Moreover, the specific mevalonate production rate exhibited a trend similar to that of cell growth: both reached a maximum during the early stage of induction and then decreased gradually. Thus, mevalonate production from acetate partially correlated with cell growth.

Mevalonate was produced by XU143 from acetate with a high maximum specific production rate (102 mg L^−1^ DCW h^−1^) compared with that achieved with glucose (50 mg L^−1^ DCW h^−1^).^1^ Therefore, XU143 may have great potential for mevalonate biosynthesis. However, low production and biomass may be attributable to 2 factors. First, acetate exerts a negative effect on intracellular protein stability.^8,9^ Second, the overexpression of heterologous genes leads to a burden on cellular metabolism. Thus, a 2-stage fermentation process was designed. The first stage produced a significant amount of biomass derived from glucose, while the second stage facilitated the accumulation of mevalonate following the addition of acetate.

### Two-stage fed-batch fermentation (FB-2)

**Figure 4.**
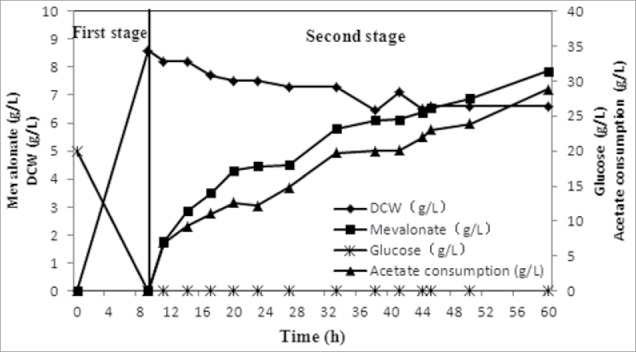
Two-stage fermentation using glucose in batch cultivation and acetate in fed-batch cultivation. When glucose was completely consumed, IPTG was added, and ammonium acetate was used as both the carbon and nitrogen source. Mevalonate concentration reached 7.85 g L^−1^, corresponding to an overall yield of 0.27 g g^−1^ and a productivity of 0.13 g L^−1^ h^−1^.

Changes in the glucose, acetate and biomass concentrations observed during the fermentation process are shown in Figure 4. Glucose was completely consumed after culture for 9 h. Then, IPTG was added, and ammonium acetate was used as both the sole carbon and nitrogen source. During the first stage of culture on glucose, the biomass concentration reached 8.6 g L^−1^ with a yield of 0.43 g of biomass g^−1^ of glucose. These values are consistent with those reported in the literature for *E. coli* grown on glucose.^14,15^ When cultivated on acetate, the *E. coli* strain appeared to adapt to this new carbon source, and the biomass decreased slightly. During this period, the mevalonate concentration increased in association with acetate consumption, meaning the strain continued to assimilate the carbon source, but carbon flux was diverted toward mevalonate synthesis, and cell proliferation halted. After cultivation for 60 h, the mevalonate concentration reached 7.85 g L^−1^, corresponding to an overall yield of 0.27 g g^−1^ and a productivity of 0.13 g L^−1^ h^−1^ (Fig. 4).

A 2-stage fermentation strategy is conducive to the bioconversion of certain products.^16,17^ However, there have been no reports describing 2-stage fermentation involving a switch in the carbon source. By using glucose during the first stage and acetate during the second stage, the mevalonate concentration and productivity greatly increased by 8.4- and 4.3-fold, respectively, compared with the results achieved in FB-1. Simultaneously, overall yield and the maximum specific mevalonate production rate resembled that of FB-1 (Table 1). This suggests that 2-stage fermentation, which avoids the limitations of acetate assimilation, is an effective strategy for mevalonate bioconversion from acetate.

**Table 1.** A comparison of mevalonate fermentation parameters among different fermentation modes.

Carbon source	Aeration	DCW_max_ (g L^−^^1^)	Mevalonate (g L^−^^1^)	q_p max_ (mg L^−^^1^ DCW h^−^^1^)	Productivity (g L^−^^1^ h^−^^1^)	Y_p_ (g g^−^^1^ acetate)
Acetate	Aerobic	0.67	1.06	102	0.03	0.30
Glucose-acetate	Aerobic	8.6	7.85	103	0.13	0.27
Glucose-acetate	Anaerobic	8.5	3.05	16.5	0.05	0.37
Glucose-acetate	Microaerobic	7.6	4.97	60	0.08	0.29

q_p max_ Maximum specific mevalonate production rate;

Productivity Biosynthesis amount of mevalonate per ferment liquid per hour;

Yield g mevalonate/ g acetate. The yield was calculated after induced only.

### Two-stage fed-batch fermentation under different aerobic conditions

**Figure 5.**
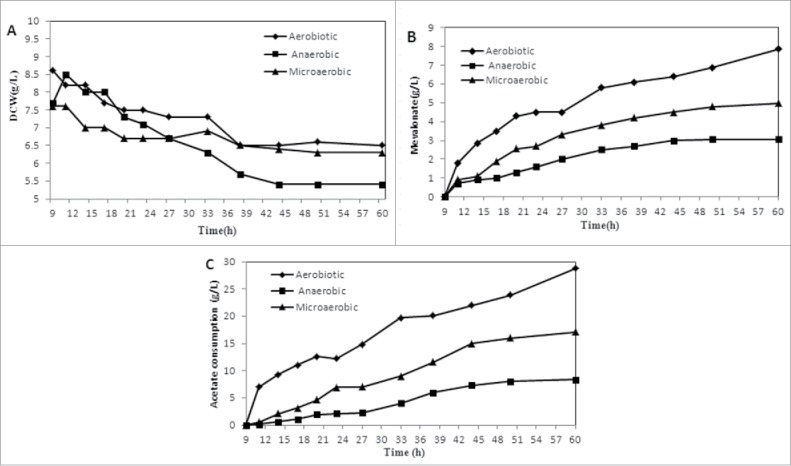
Fed-batch fermentation under different aerobic conditions. A: The cell growths; B: Mevalonate productions; C: Acetate consumptions. During the first stage, fully aerobic conditions were implemented until glucose was depleted. During the second stage, IPTG was added, and anaerobic and microaerobic conditions were implemented using ammonium acetate as the sole carbon source. This figure shows the results from the second stage of fermentation. Mevalonate concentration was 3.05 g L^−1^ with a yield of 0.37 g g^−1^ acetate under anaerobic conditions. In microaerobic conditions, mevalonate production was improved and reached 4.97 g L^−1^ with a yield of 0.29 g g^−1^ acetate at 1.0 vvm. The maximum mevalonate concentration and minimum yield were observed under fully aerobic conditions achieved with FB-2.

Aeration conditions play an important role in acetate metabolism in *E. coli*. Acetate accumulation is inversely correlated with dissolved oxygen (DO) levels when *E. coli* is grown on glucose.^18,19^ However, there have been few reports investigating acetate assimilation under different aerobic conditions. Therefore, we next performed 2-stage fed-batch fermentation under anaerobic and microaerobic (with aeration rates of 1.0 vvm) conditions. During the first stage, fully aerobic conditions were implemented until glucose was depleted. During the second stage, IPTG was added, and anaerobic and microaerobic conditions were implemented using ammonium acetate as the sole carbon source. Figure 5 shows the results from the second stage of fermentation.

Cell mass decreased in all experiments after induction, and the minimum cell mass was below 5.4 g L^−1^ under anaerobic conditions. Furthermore, the mevalonate concentration was 3.05 g L^−1^ with a yield of 0.37 g g^−1^ acetate under anaerobic conditions. Under microaerobic conditions, mevalonate production improved and reached 4.97 g L^−1^ with a yield of 0.29 g g^−1^ acetate at 1.0 vvm (Fig. 5 and Table 1). The maximum mevalonate concentration and minimum yield were observed under the fully aerobic conditions achieved with FB-2. With a lower oxygen supply, mevalonate concentration and productivity decreased, but yield increased. The distribution of catabolic flux changes under aerobic conditions, possibly to adjust the intracellular redox state.^20-22^ Therefore, the yield trend was assumed to be associated with acetate flux via the TCA and mevalonate pathways under different aerobic conditions.

## Conclusion

The engineered *E. coli* strain XU143 was capable of using acetate as a sole carbon source to produce 7.85 g L^−1^ mevalonate during a 2-stage fermentation process. Anaerobic and microaerobic fed-batch fermentation experiments indicated oxygen supply exerts a key impact on mevalonate production from acetate. Further work must be done to improve mevalonate accumulation using different feeding strategies and by optimizing culture conditions. Overall, the method described here demonstrates great potential for mevalonate production by *E. coli* using the less costly feedstock acetate.

## Materials and methods

### Strain construction

All plasmids and strains used in this study are listed in Table 2. *E. coli* W3110 genomic DNA was amplified by PCR as a template to obtain the *acs* gene using the primers *acs*-F (5′-TAGAGATCTTTACGATGGCATCGCG-3′) and *acs*-R (5′-GCCCATATGATGAGCCAAATTCACAAAC-3′). The PCR product was cloned into the pCOLADuet-1 vector between the *Bgl*II and *Nae*I sites to generate the plasmid pCOLADuet-*acs*. The *mvaE* and *mvaS* genes were cloned into pACYCDuet-1, resulting in the plasmid pYJM17 (Yang et al. 2012). pCOLADuet-1 and pACYCDuet-1 were transformed into *E. coli* BL21(DE3) to generate *E. coli* XU141. pYJM17 and pCOLADuet-1 were transformed into *E. coli* BL21(DE3) to generate *E. coli* XU142. pCOLADuet-*acs* and pYJM17 were transformed into *E. coli* BL21(DE3) to generate *E. coli* XU143.

**Table 2.** Strains and plasmids used in this study.

Strains or plasmids	Characteristic	Reference
Strains		
*E. coli* W3110	DNA template for amplification	TIANGEN
*E. coli* DH5α	Host for general cloning	TIANGEN
*E. coli* BL21 (DE3)	Host for protein expression	Novagen
XU141	*E. coli* BL21(DE3) /pCOLADuet/pACYCDuet	This study
XU142	*E. coli* BL21(DE3)/pCOLADuet/pYJM17	This study
XU143	*E. coli* BL21(DE3)/pCOLADuet-*acs*/pYJM17	This study
Plasmids		
pCOLADuet™-1	ColA origin, Kan^r^	Novagen
pACYCDuet-1	p15A (pACYC184), Cm^r^	Novagen
pCOLADuet-*acs*	pCOLADuet-1 carrying *acs* from *E. coli* W3110	This study
pYJM17	pACYCDUet-1 carrying *mvaE* and *mvaS* from *Enterococcus faecalis*	

### Medium

Primary seed cultures of XU101 were grown in LB medium containing 10 g L^−1^ tryptone, 5 g L^−1^ yeast extract, and 10 g L^−1^ NaCl. The secondary seed culture medium was the same as the fermentation medium. The fermentation minimal medium contained (per liter): 2.5 g of KH_2_PO_4_, 3 g of (NH_4_)_2_SO_4_, 1 g of citric acid·H_2_O, 1 g of C_2_H_3_O_2_Na·2H_2_O, 1.86 g of KCl, 0.08 g of FeSO_4_·7H_2_O, 0.24 g of MgSO_4_·7H_2_O, 1 g of βine, 2 g of beef powder, and 1 ml of trace elements solution. The initial carbon sources for fed-batch fermentation (acetate as the only carbon source) and 2-stage fermentation (glucose-acetate) were 2 g L^−1^ sodium acetate and 20 g L^−1^ glucose, respectively. The trace elements stock solution contained (per liter): 2.9 g of ZnSO_4_·7H_2_O, 3.7 g of (NH_4_)_6_Mo_7_O_24_·4H_2_O, 24.7 g of H_3_BO_3_, 2.5 g of CuSO_4_·5H_2_O, and 15.8 g of MnCl_2_·4H_2_O.

### Cultivation

Fed-batch fermentation was performed to scale up mevalonate production from acetate. Fermentation was performed in a Biostat B plus MO5L fermentor (Sartorius Stedim Biotech GmbH, Germany) containing 2 L of medium as described above. During the fermentation process, pH was automatically maintained at 7.0 with ammonia and sulfuric acid. During both fed-batch fermentation (acetate as the only carbon source) and 2-stage fed-batch fermentation (glucose-acetate), the dissolved oxygen (DO) concentration was maintained at 20% saturation for fully aerobic conditions. Feeding medium contained 6 M ammonium acetate. When initial glucose was depleted, ammonium acetate was fed continuously to maintain an acetate concentration ranging from 25 mmol L^−1^ to 75 mmol L^−1^ in fermentation broth. Two-stage fermentation (glucose-acetate) under microaerobic and anaerobic conditions was also performed. During the first stage, fully aerobic conditions were applied as described above until glucose was depleted, and then 6 M ammonium acetate was added followed by the implementation of either microaerobic (with aeration rates of 1.0 vvm) or anaerobic (by flushing with N_2_ instead of oxygen) conditions. Samples were withdrawn to determine cell mass and metabolite concentrations.

### Analytical methods

Biomass was determined by measuring the optical density of the culture sample at 600 nm (1 OD unit = 0.43 DCW L^−1^). The concentration of residual glucose was quantified using a SBA-40D biologic sensing analyzer. Acetate and mevalonate concentrations were determined by performing HPLC with an aminex HPX-87H ion exclusion column (Bio-Rad, USA). To analyze mevalonate, samples were prepared by mixing of 0.5 ml of 0.1-µm-filtered fermentation broth with 0.5 ml of 3 M H_2_SO_4_ and again filtering the resulting solution through a 0.1-µM filter into HPLC vials, no later than 10 min before HPLC detection.
